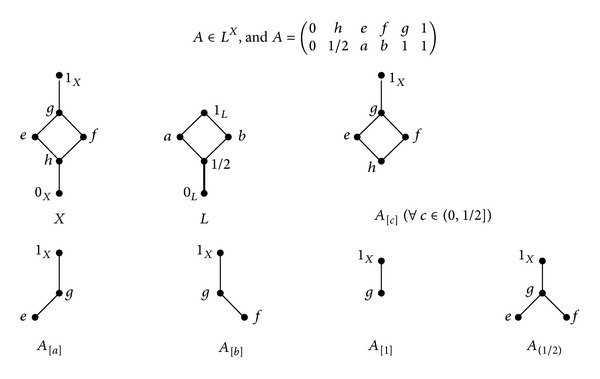# A Novel Definition of *L*-Fuzzy Lattice Based on Fuzzy Set

**DOI:** 10.1155/2013/678586

**Published:** 2013-06-05

**Authors:** Jun-Fang Zhang

**Affiliations:** Department of Basic Science, Beijing University of Agriculture, Beijing 102206, China

## Abstract

The concept of *L*-fuzzy lattice is presented by means of an *L*-fuzzy partially ordered set. An *L*-fuzzy partially ordered set *A* is an *L*-fuzzy lattice if and only if one of *A*_[*a*]_, *A* ^[*a*]^, and *A* ^(*a*)^ is a lattice.

## 1. Introduction

Many concepts of fuzzy algebra were presented, since the concept of fuzzy subgroups was introduced by Rosenfeld. The concept of fuzzy lattice was also introduced by Tepav$\overset{˘}{\text{c}}$ević and Trajkovski [1]. But the authors in [1] defined an *L*-fuzzy lattice based on a fuzzy set of a crisp lattice, so the scope is very limited, and they gave its characterizations by only one kind of its cut sets. To overcome this shortcoming, in this paper we try to present a new definition of an *L*-fuzzy lattice in an *L*-fuzzy subset of a general set in terms of an *L*-poset. Furthermore, its many characterizations are given using the theory of *L*_*β*_-sets and *L*_*α*_-sets proposed by Shi Fu-Gui in 1995. In this way, we obtain more generalized conclusion about *L*-fuzzy lattice in order to make their applications becoming comprehensive.

## 2. Preliminaries

Throughout this paper *L* denotes a completely distributive lattice, and *M*(*L*) denotes the set of all nonzero ∨-irreducible elements in *L*. *P*(*L*) denotes the set of all nonunit prime elements in *L*. *X* denotes a nonempty usual sets. *L* ^*X*^ is the set of all *L*-fuzzy sets on *X*. We will not differ a crisp set from its character function. For empty set *∅* ⊂ *L*, we define ⋀*∅* = 1 and ⋁*∅* = 0. According to [2], each element *a* in *L* has a greatest maximal family and a greatest minimal family which we, respectively, denote by *α*(*a*) and *β*(*a*). From [2] we know that α*(*a*) = *P*(*L*) ⋂ *α*(*a*) is a maximal family of *a*, *α*(1) = *∅*, and β*(*a*) = *M*(*L*) ⋂ *β*(*a*) is a minimal family of *a*, *β*(0) = *∅*.

Now we recall some basic concepts and results.

**Definition 1 (see [3])**

Let *A* ∈ *L* ^*X*^ and *a* ∈ *L*. Define

(1) A[a]={x∈X ∣ A(x)≥a},A(a)={x∈X ∣ a∈β(A(x))},A[a]={x∈X ∣ a∉α(A(x))},A(a)={x∈X ∣ A(x)≰a}.

From [3] we know that *a* ∈ *β*(*b*) implies *A*_[*b*]_ ⊂ *A*_(*a*)_ ⊂ *A*_[*a*]_ and *a* ∈ *α*(*b*) implies *A* ^[*a*]^ ⊂ *A* ^(*b*)^ ⊂ *A* ^[*b*]^. If *L* = [0,1], then *A*_[*a*]_ = *A* ^[*a*]^ and *A*_(*a*)_ = *A* ^(*a*)^.

**Definition 2 (see [3–5])**

For each *L*-fuzzy set *A* in *L* ^*X*^, we have

1. *A* = ⋁_*a*∈*L*_(*a*∧*A*_[*a*]_) = ⋁_*a*∈*M*(*L*)_(*a*∧*A*_[*a*]_) = ⋁_*a*∈*L*_(*a*∧*A*_(*a*)_) = ⋁_*a*∈*M*(*L*)_(*a*∧*A*_(*a*)_),
2. *A* = ⋀_*a*∈*L*_(*a*∨*A* ^[*a*]^) = ⋀_*a*∈*P*(*L*)_(*a*∨*A* ^[*a*]^) = ⋀_*a*∈*L*_(*a*∨*A* ^(*a*)^) = ⋀_*a*∈*P*(*L*)_(*a*∨*A* ^(*a*)^).

**Definition 3 (see [6])**

Let *A* ∈ *L* ^*X*^ and *B* ∈ *L* ^*Y*^. Define an *L*-fuzzy set *A* × *B* on *X* × *Y* by

(2) $$\begin{array}{c} \left(A\times B\right)\left(x,y\right)=A\left(x\right)\wedge B\left(y\right),\;\forall \left(x,y\right)\in X\times Y. \end{array}$$

*A* × *B* is called the product of *A* and *B*.

**Definition 4 (see [6])**

Let *A* ∈ *L* ^*X*^, *B* ∈ *L* ^*Y*^, and  *R* ∈ *L* ^*X*×*Y*^. *R* is called an *L*-fuzzy relation from (of) *A* to *B* if *R* ≤ *A* × *B*.

**Definition 5 (see [7])**

Let *A* ∈ *L* ^*X*^, *B* ∈ *L* ^*Y*^. An *L*-fuzzy relation *f* from *A* to *B* is called an *L*-fuzzy mapping from (of) *A* into *B* if *a* ∈ *M*(*L*), *f*_[*a*]_ is a mapping from (of) *A* into *B*, and then we write it as *f* : *A* → *B*.

**Theorem 6 (see [7])**

Let *A* ∈ *L* ^*X*^, *B* ∈ *L* ^*Y*^, and *f* ≤ *A* × *B*, and then the following conditions are equivalent:

1. *f* is an *L*-fuzzy mapping from *A* into *B*;
2. for each *a* ∈ *M*(*L*), *f*_(*a*)_ is a mapping from *A*_(*a*)_ into *B*_(*a*)_;
3. for each *a* ∈ *P*(*L*), *f* ^(*a*)^ is a mapping from *A* ^(*a*)^ into *B* ^(*a*)^.

**Theorem 7 (see [8])**

Let *A* ∈ *L* ^*X*^, *B* ∈ *L* ^*Y*^, and *f* ≤ *A* × *B*. If for every *b*, *c* ∈ *L*, α*(*b*∨*c*) = α*(*b*)⋂α*(*c*), then the following conditions are equivalent:

1. *f* is an *L*-fuzzy mapping from *A* into *B*;
2. for every *a* ∈ α*(0), *f* ^[*a*]^ is a mapping from *A* ^[*a*]^ into *B* ^[*a*]^.

**Definition 8 (see [9])**

Let *A* ∈ *L* ^*X*^, *B* ∈ *L* ^*Y*^, and *f* : *A* → *B*. For *C* ≤ *A*, we define *f*(*C*) = ⋁_*a*∈*M*(*L*)_(*a*∧*f*_[*a*]_(*C*_[*a*]_)). Then *f*(*C*) is called the image of *C* under *f*.

**Theorem 9 (see [9])**

Let *A* ∈ *L* ^*X*^, *B* ∈ *L* ^*Y*^, and *f* : *A* → *B*, and then for *C* ≤ *A* we have

1. for each *a* ∈ *M*(*L*), *f*(*C*)_(*a*)_⊆*f*_(*a*)_(*C*_(*a*)_)⊆*f*_[*a*]_(*C*_[*a*]_)⊆*f*(*C*)_[*a*]_,
2. *f*(*C*) = ⋁_*a*∈*M*(*L*)_(*a*∧*f*_(*a*)_(*C*_(*a*)_)),
3. for each *a* ∈ *P*(*L*), *f*(*C*)^(*a*)^ = *f* ^(*a*)^(*C* ^(*a*)^),
4. *f*(*C*) = ⋀_*a*∈*P*(*L*)_(*a*∨*f* ^(*a*)^(*C* ^(*a*)^)).

**Definition 10 (see [9])**

Let *A* ∈ *L* ^*X*^, *B* ∈ *L* ^*Y*^, and *f* : *A* → *B*. For *D* ≤ *B*, we define *f* ^−1^(*D*) = ⋁_*a*∈*M*(*L*)_(*a*∧(*f*_[*a*]_)^−1^(*D*_[*a*]_)). Then *f* ^−1^(*D*) is called the inverse image of *D* under *f*.

**Theorem 11 (see [9])**

Let *A* ∈ *L* ^*X*^, *B* ∈ *L* ^*Y*^, and *f* : *A* → *B*. Then for *D* ≤ *B*, we have

1. for each *a* ∈ *M*(*L*), (*f* ^−1^(*D*))_(*a*)_⊆(*f*_(*a*)_)^−1^(*D*_(*a*)_)⊆(*f*_[*a*]_)^−1^(*D*_[*a*]_)⊆(*f* ^−1^(*D*))_[*a*]_,
2. *f* ^−1^(*D*) = ⋁_*a*∈*M*(*L*)_(*a*∧(*f*_(*a*)_)^−1^(*D*_(*a*)_)),
3. for each *a* ∈ *P*(*L*), (*f* ^−1^(*D*))^(*a*)^ = (*f* ^(*a*)^)^−1^(*D* ^(*a*)^),
4. *f* ^−1^(*D*) = ⋀_*a*∈*P*(*L*)_(*a*∨(*f* ^(*a*)^)^−1^(*D* ^(*a*)^)).

**Definition 12 (see [10])**

Let *X* be a set, *A* ∈ *L* ^*X*^, and *A* ≠ 0. An *L*-fuzzy relation *R* from *A* to *A* is called an *L*-fuzzy partial order on *A* if *R* satisfies the following conditions:

1. for all *x* ∈ *A*_(0)_, *R*(*x*, *x*) = *A*(*x*),
2. *R*∘*R* ≤ *R*,
3. for all *x*, *y* ∈ *A*_(0)_, *R*(*x*, *y*)∧*R*(*y*, *x*) ≠ 0⇒*x* = *y*.

When *R* is an *L*-fuzzy partial order on *A*, we call (*A*, *R*) an *L*-fuzzy partial order set or *L*-poset for short.

**Theorem 13 (see [10])**

For an *L*-fuzzy relation *R* on *A*, the following implications (4)⇒(1) and (1)⇔(2)⇔(3)⇔(5)⇔(6)⇔(7) are true.

1. *R* is an *L*-fuzzy partial order on *A*.
2. For each *a* ∈ *L*∖_{0}_, if *R*_[*a*]_ is not empty set, then it is a partial order on *A*_[*a*]_.
3. For each *a* ∈ *M*(*L*), if *R*_[*a*]_ is not empty set, then it is a partial order on *A*_[*a*]_.
4. For each *a* ∈ *β*(1), if *R*_(*a*)_ is not empty set, then it is a partial order on *A*_(*a*)_.
5. For each *a* ∈ *α*(0), if *R* ^[*a*]^ is not empty set, then it is a partial order on *A* ^[*a*]^.
6. For each *a* ∈ α*(0), if *R* ^[*a*]^ is not empty set, then it is a partial order on *A* ^[*a*]^.
7. For each *a* ∈ *P*(*L*), if *R* ^(*a*)^ is not empty set, then it is a partial order on *A* ^(*a*)^.

**Remark 14 (see [10])**

In general, (1)⇒(4) in the previous theorem is not true. This can be seen from the following example.

**Example 15 (see [10])**

Let *X* = {*x*, *y*, *z*},  *L* = {1, *a*, *b*, *c*}∪[0, 1/2], where *x*, *y*, and  *z* are different and [0, 1/2] is an interval. We define the order in *L* as follow.

For all *e* ∈ [0, 1/2],  *e* ≤ *c* = 1/2,  *c* < *a*,  *c* < *b*,  *a*≰*b*,  *b*≰*a*,  *a* < 1,  and  *b* < 1. The order in [0, 1/2] is as usual. Then *L* is a completely distributive lattice. Take *R* ∈ *L* ^*X*×*X*^ such that

(3) $$\begin{array}{c} R\left(x,x\right)=R\left(y,y\right)=R\left(z,z\right)=1,\\ R\left(y,x\right)=R\left(z,y\right)=R\left(z,x\right)=0,\\ R\left(x,y\right)=a,\;\;R\left(y,z\right)=b,\\ R\left(x,z\right)=\frac{1}{2}. \end{array}$$

Obviously we have

(4) $$\begin{array}{c} \left(R\circ R\right)\left(x,x\right)=\left(R\circ R\right)\left(y,y\right)=\left(R\circ R\right)\left(z,z\right)=1,\\ \left(R\circ R\right)\left(z,y\right)=\left(R\circ R\right)\left(y,x\right)=\left(R\circ R\right)\left(z,x\right)=0,\\ \left(R\circ R\right)\left(x,y\right)=a,\;\;\left(R\circ R\right)\left(y,z\right)=b,\\ \left(R\circ R\right)\left(x,z\right)=\frac{1}{2}. \end{array}$$

Thus *R* is an *L*-fuzzy partial order on *X*. But it is easy to check that

(5) R(1/2)={(x,x),(y,y),(z,z),(x,y),(y,z)},R(1/2)∘R(1/2)={(x,x),(y,y),(z,z),(x,y),(y,z),(x,z)}⊄R(1/2).

This shows that *R*_(1/2)_ is not a partial order on *X*.

## 3. *L*-Fuzzy Lattice

**Definition 16**

Let *A* ∈ *L* ^*X*^, *x*, *y*, *s* ∈ *X*, *R* be an *L*-fuzzy partial order on *A*. *s* is called an *L*-fuzzy supremum of *x*, *y* if the following conditions are true:

(S1) *A*(*s*) ≥ *A*(*x*)∧*A*(*y*)
(S2) *R*(*x*, *s*) ≥ *R*(*x*, *x*);  *R*(*y*, *s*) ≥ *R*(*y*, *y*),
(S3) *R*(*s*, *z*) ≥ *R*(*x*, *z*)∧*R*(*y*, *z*).

**Definition 17**

Let *A* ∈ *L* ^*X*^, *x*, *y*, *t* ∈ *X*, *R* be an *L*-fuzzy partial order on *A*. *t* is called an *L*-fuzzy infimum of *x*, *y* if the following conditions are true:

(T1) *A*(*t*) ≥ *A*(*x*)∧*A*(*y*),
(T2) *R*(*t*, *x*) ≥ *R*(*x*, *x*);  *R*(*t*, *y*) ≥ *R*(*y*, *y*),
(T3) *R*(*z*, *t*) ≥ *R*(*z*, *x*)∧*R*(*z*, *y*).

**Definition 18**

An *L*-fuzzy partially ordered set (*A*, *R*) is called an *L*-fuzzy lattice on *X* if for any *x*, *y* ∈ *A*_(0)_, both *L*-fuzzy supremum and *L*-fuzzy infimum of *x*, *y* exist.

**Theorem 19**

Let (*A*, *R*) be an *L*-fuzzy partially ordered set. Then the following conditions are equivalent.

1. (*A*, *R*) is an *L*-fuzzy lattice on *X*.
2. For any *a* ∈ *L*∖_{0}_, (*A*_[*a*]_, *R*_[*a*]_) is a lattice.
3. For any *a* ∈ *M*(*L*), (*A*_[*a*]_, *R*_[*a*]_) is a lattice.
4. For any *a* ∈ *α*(0), (*A* ^[*a*]^, *R* ^[*a*]^) is a lattice.
5. For any *a* ∈ α*(0), (*A* ^[*a*]^, *R* ^[*a*]^) is a lattice.
6. For any *a* ∈ *P*(*L*), (*A* ^(*a*)^, *R* ^(*a*)^) is a lattice.

**Proof**

(1)⇒(2)⇒(3)⇒(6)⇒(1). Since above-mentioned sets have been posets, we only need to prove that supremum and infimum of *x*, *y* exist.

(1)⇒(2). For any *a* ∈ *L*∖_{0}_, let *x*, *y* ∈ *A*_(0)_ and *x*, *y* ∈ *A*_[*a*]_, and then *A*(*x*) ≥ *a*, *A*(*y*) ≥ *a*. By Definitions 1 and 2 we know that there exist *s*, *t* ∈ *A*_(0)_ such that

(6) $$\begin{array}{c} A\left(S\right)\geq A\left(x\right)\wedge A\left(y\right)\geq a,\;\;A\left(I\right)\geq A\left(x\right)\wedge A\left(y\right)\geq a. \end{array}$$

So *s*, *t* ∈ *A*_[*a*]_. By (S2) we know that *R*(*x*, *s*) ≥ *R*(*x*, *x*) = *A*(*x*) ≥ *a* and *R*(*y*, *s*) ≥ *R*(*y*, *y*) = *A*(*y*) ≥ *a*. Therefore, (*x*, *s*) ∈ *R*_[*a*]_, (*y*, *s*) ∈ *R*_[*a*]_. Analogously we can prove that (*t*, *x*) ∈ *R*_[*a*]_, (*t*, *y*) ∈ *R*_[*a*]_. For any (*x*, *z*), (*y*, *z*) ∈ *R*_[*a*]_, we have that *R*(*s*, *z*) ≥ *R*(*x*, *z*)∧*R*(*y*, *z*) ≥ *a*, hence (*s*, *z*) ∈ *R*_[*a*]_. Analogously, for any (*z*, *x*), (*z*, *y*) ∈ *R*_[*a*]_, we can prove that (*z*, *t*) ∈ *R*_[*a*]_. Thereby, *s*, *t* are, respectively, supremum and infimum of *x*, *y* with respect to *R*_[*a*]_ in *A*_[*a*]_. Then it is proved that (*A*_[*a*]_, *R*_[*a*]_) is a lattice.

(2)⇒(3) is obvious.

(3)⇒(6). For any *a* ∈ *P*(*L*), let *x*, *y* ∈ *A* ^(*a*)^. Since *A* ^(*a*)^ = ⋃_*b*≰*a*_ *A*_[*b*]_, there exist *b*_1_, *b*_2_≰*a* such that *x* ∈ *A*_[*b*_1_]_, *y* ∈ *A*_[*b*_2_]_. Take *b* = *b*_1_∧*b*_2_, then we have *x*, *y* ∈ *A*_[*b*]_ and *b* = *b*_1_∧*b*_2_≰*a* (since a is a prime element). By (3) we know that for any *a* ∈ *M*(*L*), (*A*_[*a*]_, *R*_[*a*]_) is a lattice. Then there exist *x* ∨_*b*_ *y*, *x* ∧_*b*_ *y* ∈ *A*_[*b*]_ ⊂ *A* ^(*a*)^ such that

(7) $$\begin{array}{c} \left(x,x\;\vee_{b}\;y\right)\in R_{\left[b\right]}\subset R^{\left(a\right)},\;\;\left(y,x\;\vee_{b}\;y\right)\in R_{\left[b\right]}\subset R^{\left(a\right)},\\ \forall \left(x,z\right),\left(y,z\right)\in R_{\left[b\right]}\subset R^{\left(a\right)},\;\;\left(x\;\vee_{b}\;y,z\right)\in R_{\left[b\right]}\subset R^{\left(a\right)}. \end{array}$$

Thus *x* ∨_*b*_ *y* is supremum with respect to *R* ^(*a*)^ of *x*, *y* in *A* ^(*a*)^. Analogously we can prove that infimum exists in *A* ^(*a*)^ too. Hence (*A* ^(*a*)^, *R* ^(*a*)^) is a lattice.

(6)⇒(1). Let *x*, *y* ∈ *A*_(0)_ and *a* ∈ *P*(*L*),  *A*(*x*)∧*A*(*y*)≰*a*. Then *A*(*x*)≰*a*,  *A*(*y*)≰*a*; that is, *x*, *y* ∈ *A* ^(*a*)^. From (6) there exist *x* ∨_*a*_ *y* and *x* ∧_*a*_ *y* in *A* ^(*a*)^; that is, *A*(*x* ∨_*a*_ *y*)≰*a* and *A*(*x* ∧_*a*_ *y*)≰*a*. So we have

(8) $$\begin{array}{c} A\left(x\;\vee_{a}\;y\right)\geq A\left(x\right)\wedge A\left(y\right),\;\;A\left(x\;\wedge_{a}\;y\right)\geq A\left(x\right)\wedge A\left(y\right) \end{array}$$

and by (6) we obtain that (*x*, *x* ∨_*a*_ *y*) ∈ *R* ^(*a*)^ and (*y*, *x* ∨_*a*_ *y*) ∈ *R* ^(*a*)^. This shows that *R*(*x*, *x* ∨_*a*_ *y*)≰*a*, *R*(*y*, *x* ∨_*a*_ *y*)≰*a*. Therefore,

(9) R(x,x ∨a y)≥A(x)=R(x,x),R(y,x ∨a y)≥A(y)=R(y,y).

By (6) we have that for each (*x*, *z*), (*y*, *z*) ∈ *R* ^(*a*)^; that is, *R*(*x*, *z*)≰*a*, *R*(*y*, *z*)≰*a*, and we can obtain that (*x* ∨_*a*_ *y*, *z*) ∈ *R* ^(*a*)^; that is, *R*(*x* ∨_*a*_ *y*, *z*)≰*a*. Therefore *R*(*x* ∨_*a*_ *y*, *z*) ≥ *R*(*x*, *z*)∧*R*(*y*, *z*). As before we can prove that

(10) $$\begin{array}{c} R\left(x\;\wedge_{a}\;y,x\right)\geq R\left(x,x\right),\;\;R\left(x\;\wedge_{a}\;y,y\right)\geq R\left(y,y\right),\\ R\left(z,x\;\wedge_{a}\;y\right)\geq R\left(z,x\right)\wedge R\left(z,y\right). \end{array}$$

Let *s* = *x* ∨_*a*_ *y*, *t* = *x* ∧_*a*_ *y*. Then from Definition 1 we know that *s*, *t* are *L*-supremum and *L*-infimum of *x*, *y* respectively.

Then we prove (1)⇒(4)⇒(5)⇒(1).

(1)⇒(4). For any *a* ∈ α*(0), let *x*, *y* ∈ *A* ^[*a*]^; that is, *a* ∉ *α*(*A*(*x*)), *a* ∉ *α*(*A*(*y*)). From (1) there exist *s*, *t* such that *A*(*s*) ≥ *A*(*x*)∧*A*(*y*) and *A*(*t*) ≥ *A*(*x*)∧*A*(*y*). This implies that *a* ∉ *α*(*A*(*s*)) and *a* ∉ *α*(*A*(*t*)); that is, *s*, *t* ∈ *A* ^[*a*]^. From (1) we know that *R*(*x*, *s*) ≥ *R*(*x*, *x*) = *A*(*x*) and *R*(*y*, *s*) ≥ *R*(*y*, *y*) = *A*(*y*). Then we obtain that

(11) a∉α(R(x,s)), a∉α(R(x,t)),that is  (x,s)∈R[a], (y,s)∈R[a].

And by (S2) : *R*(*s*, *z*) ≥ *R*(*x*, *z*)∧*R*(*y*, *z*) we have that for each (*x*, *z*), (*y*, *z*) ∈ *R* ^[*a*]^; that is, *a* ∉ *α*(*R*(*x*, *z*)), *a* ∉ *α*(*R*(*y*, *z*)), and then *a* ∉ *α*(*R*(*s*, *z*)); that is, (*s*, *z*) ∈ *R* ^[*a*]^. Analogously we prove that for any (*z*, *x*), (*z*, *y*) ∈ *R* ^[*a*]^, (*z*, *t*) ∈ *R* ^[*a*]^. Hence *s* is supremum of *x*, *y* with respect to *R* ^[*a*]^ in *A* ^[*a*]^. Similarly we can prove that *t* is infimum of *x*, *y* with respect to *R* ^[*a*]^ in *A* ^[*a*]^. So (*A* ^[*a*]^, *R* ^[*a*]^) is a lattice.

(4)⇒(5) is obvious.

(5)⇒(1). For any *x*, *y* ∈ *A*_(0)_, let *a* ∈ α*(0) and *a* ∉ *α*(*A*(*x*)), *a* ∉ *α*(*A*(*y*)); that is, *x*, *y* ∈ *A* ^[*a*]^. From (5) there exist *x* ∨_*a*_ *y*, *x* ∧_*a*_ *y* ∈ *A* ^[*a*]^; that is, *a* ∉ *α*(*A*(*x* ∨_*a*_ *y*)), *a* ∉ *α*(*A*(*x* ∧_*a*_ *y*)). This shows that

(12) $$\begin{array}{c} A\left(x\;\vee_{a}\;y\right)\geq A\left(x\right)\wedge A\left(y\right),\\ A\left(x\;\wedge_{a}\;y\right)\geq A\left(x\right)\wedge A\left(y\right). \end{array}$$

From (5) we have that (*x*, *x* ∨_*a*_ *y*) ∈ *R* ^[*a*]^ and (*y*, *x* ∨_*a*_ *y*) ∈ *R* ^[*a*]^; that is, *a* ∉ *α*(*R*(*x*, *x* ∨_*a*_ *y*)) and *a* ∉ *α*(*R*(*y*, *x* ∨_*a*_ *y*)). Therefore,

(13) $$\begin{array}{c} R\left(x,x\;\vee_{a}\;y\right)\geq A\left(x\right)=R\left(x,x\right),\\ R\left(y,x\;\vee_{a}\;y\right)\geq A\left(y\right)=R\left(y,y\right). \end{array}$$

And by (5), for any (*x*, *z*), (*y*, *z*) ∈ *R* ^[*a*]^; that is, *a* ∉ *α*(*R*(*x*, *z*)), *a* ∉ *α*(*R*(*y*, *z*)). Then we have (*x* ∨_*a*_ *y*, *z*) ∈ *R* ^[*a*]^; that is, *a* ∉ *α*(*R*(*x* ∨_*a*_ *y*, *z*)). Hence *R*(*x* ∨_*a*_ *y*, *z*) ≥ *R*(*x*, *z*)∧*R*(*y*, *z*). Analogously we can conclude that

(14) $$\begin{array}{c} R\left(x\;\wedge_{a}\;y,x\right)\geq R\left(x,x\right),\\ R\left(x\;\wedge_{a}\;y,y\right)\geq R\left(y,y\right),\\ R\left(z,x\;\wedge_{a}\;y\right)\geq R\left(z,x\right)\wedge R\left(z,y\right). \end{array}$$

Let *s* = *x* ∨_*a*_ *y*, *t* = *x* ∧_*a*_ *y*, and then *s*, *t* are *L*-supremum and *L*-infimum of *x*, *y*, respectively.

**Theorem 20**

Let (*A*, *R*) be an *L*-fuzzy partially ordered set. If for each *a* ∈ β*(1),(*A*_(*a*)_, *R*_(*a*)_) is a lattice, and then (*A*, *R*) is an *L*-fuzzy lattice on *X*.

**Proof**

For any *x*, *y* ∈ *A*_(0)_, let *a* ∈ β*(1),  *a* ∈ *β*(*A*(*x*)) and *a* ∈ *β*(*A*(*y*)); that is, *x*, *y* ∈ *A*_(*a*)_. There exist *x* ∨_*a*_ *y* and *x* ∧_*a*_ *y*∈; that is, *a* ∈ *β*(*A*(*x* ∨_*a*_ *y*)) and *a* ∈ *β*(*A*(*x* ∧_*a*_ *y*)). Thereby

(15) $$\begin{array}{c} A\left(x\;\vee_{a}\;y\right)\geq A\left(x\right)\wedge A\left(y\right),\\ A\left(x\;\wedge_{a}\;y\right)\geq A\left(x\right)\wedge A\left(y\right). \end{array}$$

Since (*A*_(*a*)_, *R*_(*a*)_) is a lattice, we have that (*x*, *x* ∨_*a*_ *y*) ∈ *R*_(*a*)_ and (*y*, *x* ∨_*a*_ *y*) ∈ *R*_(*a*)_; that is, *a* ∈ *β*(*R*(*x*, *x* ∨_*a*_ *y*)) and *a* ∈ *β*(*R*(*y*, *x* ∨_*a*_ *y*)). This implies that

(16) R(x,x ∨a y)≥A(x)=R(x,x),R(y,x ∨a y)≥A(y)=R(y,y).

Analogously we can conclude that for all (*x*, *z*), (*y*, *z*) ∈ *R*_(*a*)_; that is, *a* ∈ *β*(*R*(*x*, *z*)), *a* ∈ *β*(*R*(*y*, *z*)), and we have (*x* ∨_*a*_ *y*, *z*) ∈ *R*_(*a*)_; that is, *a* ∈ *β*(*R*(*x* ∨_*a*_ *y*, *z*)). Then we obtain that *R*(*x* ∨_*a*_ *y*, *z*) ≥ *R*(*x*, *z*)∧*R*(*y*, *z*). Similarly, we can prove that

(17) $$\begin{array}{c} R\left(x\;\wedge_{a}\;y,x\right)\geq R\left(x,x\right),\\ R\left(x\;\wedge_{a}\;y,y\right)\geq R\left(y,y\right),\\ R\left(z,x\;\wedge_{a}\;y\right)\geq R\left(z,x\right)\wedge R\left(z,y\right). \end{array}$$

Let *s* = *x* ∨_*a*_ *y*, *t* = *x* ∧_*a*_ *y*, so it is proved that *s*, *t* are *L*-supremum and *L*-infimum of *x*, *y*, respectively.

**Remark 21**

Inversely the previous theorem is not true when (*A*, *R*) is a poset. This can be seen from Remark 14.

**Definition 22**

Let *X* be a nonempty set, and let *A*, *B* be *L*-fuzzy lattices of *X*. *B* is called an *L*-fuzzy sublattice of *A* if *B* ≤ *A*.

**Definition 23**

Let *X*, *Y* be nonempty sets, and let *A*, *B* be *L*-fuzzy lattices of *X*, *Y*. An *L*-fuzzy mapping *f* : *A* → *B* is called an *L*-fuzzy lattice homomorphism if for any *a* ∈ *P*(*L*), *f* ^(*a*)^ : *A* ^(*a*)^ → *B* ^(*a*)^ is a lattice homomorphism.

From the corresponding theorems in [9] and knowledge in general algebra we can easily obtain the following theorem.

**Theorem 24**

Let *X*, *Y* be nonempty sets, let *A*, *B* be *L*-fuzzy lattices of *X*, *Y*, let *f* : *A* → *B* be an *L*-fuzzy lattice homomorphism, and then the following propositions are true.

1. If *C* is an *L*-fuzzy sublattice of *A*, then *f*(*C*) is an *L*-fuzzy sublattice of *B*.
2. If *D* is an *L*-fuzzy sublattice of *B*, then *f* ^−1^(*D*) is an *L*-fuzzy sublattice of *A*.

## 4. Fuzzy Sublattice

In [1], the author gave an *L*-valued fuzzy lattice. From the following analysis we can see that *L*-valued fuzzy lattice is a special case of bifuzzy lattices in fact.

**Definition 25 (see [1])**

Let *L* be a complete lattice with the greatest element 1_*L*_ and the least element 0_*L*_, and let (*X*, *R*) be a lattice,   *A* ∈ *L* ^*X*^.  *A* is called a lattice-valued fuzzy lattice if all the *p*-cuts of *A* are sublattices of *X*.

**Remark 26**

Here the *p*-cut of *A* indicates the case of *A*_[*a*]_ in fact.

**Theorem 27 (see [1])**

Let *L* be a complete lattice with the greatest element 1_*L*_ and the least element 0_*L*_, let (*X*, *R*) be a lattice, *A* ∈ *L* ^*X*^, and then *A* is called an L-valued fuzzy lattice if and only if for all *x*, *y* ∈ *X*, the following conditions are true:

(A1) *A*(*x*∨*y*) ≥ *A*(*x*)∧*A*(*y*),
(A2) *A*(*x*∧*y*) ≥ *A*(*x*)∧*A*(*y*).

**Remark 28**

When *L* is a completely distributive lattice, (*X*, *R*) is a lattice, here *R* is a relation on *X*. In the Definitions 16–18 of *L*-fuzzy lattice, let *L*-fuzzy partial order *R*′ = *R*∩(*A* × *A*)⊆*A* × *A*, *R*′ = *R*∩(*A* × *A*)⊆*A* × *A*, and then the conditions (R1)–(R4) in Definition 25 are true. Thus it can be seen that the *L*-valued fuzzy lattice is a special case of *L*-fuzzy lattice. Since *A* is an fuzzy subset of *X*, we call this *L*-valued fuzzy lattice *A* as fuzzy sublattice of *X* in the following application.

Now we study the relation between fuzzy sublattice and crisp lattice by means of their four level cut sets, furthermore we give the definition of fuzzy lattice homomorphism and corresponding theorems.

**Definition 29**

Let *L* be a completely distributive lattice, let (*X*, *R*) be a lattice, and let *R* be a relation on *X*, *A* ∈ *L* ^*X*^, if for all *x*, *y* ∈ *X*, there exist *x*∨*y*, *x*∧*y* ∈ *X* such that

(A1) *A*(*x*∨*y*) ≥ *A*(*x*)∧*A*(*y*),
(A2) *A*(*x*∧*y*) ≥ *A*(*x*)∧*A*(*y*), then we call *A* as a fuzzy sublattice of *X*.

**Theorem 30**

Let *L* be a completely distributive lattice, let (*X*, *R*) be a lattice, and let *R* be a relation on *X*, *A* ∈ *L* ^*X*^. Then we can obtain that (1), (2), (3), (4), (5),  and  (8) are equivalent, and (6)⇒(1) is true.

1. *A* is a fuzzy sublattice of *X*.
2. For each *a* ∈ *L*, (*A*_[*a*]_, *R*) is a sublattice of (*X*, *R*).
3. For each *a* ∈ *M*(*L*), (*A*_[*a*]_, *R*) is a sublattice of (*X*, *R*).
4. For each *a* ∈ *L*, (*A* ^[*a*]^, *R*) is a sublattice of (*X*, *R*).
5. For each *a* ∈ *P*(*L*), (*A* ^[*a*]^, *R*) is a sublattice of (*X*, *R*).
6. For each *a* ∈ *L*, (*A*_(*a*)_, *R*) is a sublattice of (*X*, *R*).
7. For each *a* ∈ *M*(*L*), (*A*_(*a*)_, *R*) is a sublattice of (*X*, *R*).
8. For each *a* ∈ *P*(*L*), (*A* ^(*a*)^, *R*) is a sublattice of (*X*, *R*).

**Proof**

(1)⇒(2). For each *a* ∈ *L*, let *x*, *y* ∈ *A*_[*a*]_. Then we have *A*(*x*) ≥ *a*, *A*(*y*) ≥ *a*. From (1), there exist *x*∨*y*, *x*∧*y* ∈ *X* such that *A*(*x*∨*y*) ≥ *A*(*x*)∧*A*(*y*) ≥ *a* and *A*(*x*∧*y*) ≥ *A*(*x*)∧*A*(*y*) ≥ *a*. Therefore *x*∨*y*, *x*∧*y* ∈ *A*_[*a*]_. Then it is proved that (*A*_[*a*]_, *R*) is a sublattice of (*X*, *R*).

(2)⇒(3) is obvious.

(3)⇒(8). For each *a* ∈ *P*(*L*), let x,y∈A(a)=⋃b≰ab∈M(L)A[b]. There exist *b*_1_≰*a*, *b*_2_≰*a* such that *x* ∈ *A*_[*b*_1_]_, *y* ∈ *A*_[*b*_2_]_. Hence *x*, *y* ∈ *A*_[*b*_1_∧*b*_2_]_ = *A*_[*b*]_. Since *a* is a prime element, we have that *b* = *b*_1_∧*b*_2_≰*a*. From (3), we have *x*∨*y*, *x*∧*y* ∈ *A*_[*b*]_, therefore *x*∨*y*, *x*∧*y* ∈ *A* ^(*a*)^. So we obtain that (*A* ^(*a*)^, *R*) is a sublattice of (*X*, *R*).

(8)⇒(4). For each *a* ∈ *L*, let *x*, *y* ∈ *A* ^[*a*]^ = ⋂_*b*∈*α*(*a*)_ *A* ^(*b*)^. Hence for all *b* ∈ *α*(*a*), and *b* is a prime element. We know that *x*, *y* ∈ *A* ^(*b*)^. From (8), (*A* ^(*b*)^, *R*) is a sublattice of (*X*, *R*), so we have *x*∨*y*, *x*∧*y* ∈ *A* ^(*b*)^. Thus *x*∨*y*, *x*∧*y* ∈ *A* ^[*a*]^. Therefore, (*A* ^[*a*]^, *R*) is a sublattice of (*X*, *R*).

(4)⇒(5) is obvious.

(5)⇒(1). For each *x*, *y* ∈ *X*, let *a* ∈ *P*(*L*) and *x*, *y* ∈ *A* ^[*a*]^; that is, *a* ∉ *α*(*A*(*x*)), *a* ∉ *α*(*A*(*y*)). From (5) there exist *x*∨*y*, *x*∧*y* ∈ *A* ^[*a*]^; that is, *a* ∉ *α*(*A*(*x*∨*y*)), *a* ∉ *α*(*A*(*x*∧*y*)). Thus *A*(*x*∨*y*) ≥ *A*(*x*)∧*A*(*y*), *A*(*x*∧*y*) ≥ *A*(*x*)∧*A*(*y*). Therefore, *A* is a fuzzy lattice of *X*.

(6)⇒(7) is obvious.

(7)⇒(1). For each *x*, *y* ∈ *X*, let *a* ∈ *M*(*L*) and *x*, *y* ∈ *A*_(*a*)_; that is, *a* ∈ *β*(*A*(*x*)), *a* ∈ *β*(*A*(*y*)). From (7), there exist *x*∨*y*, *x*∧*y* ∈ *A*_(*a*)_; that is, *a* ∈ *β*(*A*(*x*∨*y*)), *a* ∈ *β*(*A*(*x*∧*y*)). So it is proved that *A*(*x*∨*y*) ≥ *A*(*x*)∧*A*(*y*) and *A*(*x*∧*y*) ≥ *A*(*x*)∧*A*(*y*). This shows that *A* is a fuzzy lattice of *X*.

**Remark 31**

Generally, (1)⇒(6) in the previous theorem is not true. This can be seen from the following example.

**Example 32**

*X* = {0_*X*_, *h*, *e*, *f*, *g*, 1_*X*_}, where *e*≰*f*, *f*≰*e*.   *L* = [0_*L*_, 1/2]∪{*a*, *b*, 1_*X*_}, where *a*≰*b*, *b*≰*a*.  *A* ∈ *L* ^*X*^ (see Figure 1).

Then *A*_[0_*L*_]_ = *X*,  for all  *c* ∈ (0_*L*_, 1/2], *A*_[*c*]_ = {*h*, *e*, *f*, *g*, 1_*X*_}, *A*_[*a*]_ = {*e*, *g*, 1_*X*_}, *A*_[*b*]_ = {*f*, *g*, 1_*X*_}, and *A*_[1_*L*_]_ = {*g*, 1_*X*_}. This implies for all *a* ∈ *L*, (*A*_[*a*]_, *R*) is a sublattice of *X*; that is, *A* is a fuzzy lattice of *X*. While for 1/2 ∈ β*(1), *A*_(1/2)_ = {*e*, *f*, *g*, 1_*X*_} is not a sublattice of *X*. This implies that (1)⇏(6).

**Remark 33**

The following condition makes (1)⇒(6) be true.

**Theorem 34**

If for all *b*, *c* ∈ *L*, *β*(*b*∧*c*) = *β*(*b*) ⋂ *β*(*c*), then (1)⇒(6) in the previous theorem is true.

**Proof**

(1)⇒(6). For each *a* ∈ *L*, *x*, *y* ∈ *A*_(*a*)_, and then *a* ∈ *β*(*A*(*x*)), *a* ∈ *β*(*A*(*y*)). Therefore, *a* ∈ *β*(*A*(*x*)∧*A*(*y*)) = *β*(*A*(*x*))∩*β*(*A*(*y*)). From (1) we know that *A*(*x*∨*y*) ≥ *A*(*x*)∧*A*(*y*) and *A*(*x*∧*y*) ≥ *A*(*x*)∧*A*(*y*). So we have that *a* ∈ *β*(*A*(*x*∨*y*)) and *a* ∈ *β*(*A*(*x*∧*y*)). This shows that (*A*_(*a*)_, *R*) is a sublattice of (*X*, *R*).

**Definition 35**

Let *X* be a nonempty set, let *A*, *B* be fuzzy lattices of *X*, and if *B* ≤ *A*, we call *B* as a fuzzy sublattice of *A*.

**Definition 36**

Let *X*, *Y* be nonempty sets, and let *A*, *B* be fuzzy sublattices of *X*, *Y*, respectively. *L*-fuzzy mapping *f* : *A* → *B* is named a fuzzy lattice homomorphism if it satisfies that for all *a* ∈ *P*(*L*), *f* ^(*a*)^ : *A* ^(*a*)^ → *B* ^(*a*)^ is a lattice homomorphism.

The following theorems are easily obtained by the corresponding theorem in [9] and the knowledge in general algebra.

**Theorem 37**

Let *X*, *Y* be nonempty sets, and let *A*, *B* be fuzzy sublattices of *X*, *Y*, respectively. *f* : *A* → *B* is a fuzzy lattice homomorphism, and then the following conditions are true:

1. if *C* is a fuzzy sublattice of *A*, then *f*(*C*) is a fuzzy sublattice of *B*;
2. if *D* is a fuzzy sublattice of *B*, then *f* ^−1^(*D*) is a fuzzy sublattice of *A*.

**Theorem 38**

Let *X*, *Y* be nonempty sets, and let *A*, *B* be fuzzy sublattices of *X*, *Y*, respectively. *f* : *A* → *B* is a fuzzy mapping, then the following conditions are true:

1. *f* : *A* → *B* is a fuzzy lattice homomorphism;
2. for all *a* ∈ *M*(*L*), *f*_[*a*]_ is a lattice homomorphism from *A*_[*a*]_ to *B*_[*a*]_.

**Proof**

(1)⇒(2). Let *a* ∈ *M*(*L*) and *x*, *y* ∈ *A*_[*a*]_, and then *A*(*x*) ≥ *a*, *A*(*y*) ≥ *a*. For any *b* ∈ *P*(*L*), *b*≱*a*, we have *x* ∈ *A* ^(*b*)^, *y* ∈ *A* ^(*b*)^. From (1) we know that *f* : *A* → *B* is a fuzzy lattice homomorphism. Thus there exist *u*_*b*_ ∈ *B* ^(*b*)^, *v*_*b*_ ∈ *B* ^(*b*)^ such that (*x*, *u*_*b*_) ∈ *f* ^(*b*)^, (*y*, *v*_*b*_) ∈ *f* ^(*b*)^; that is, *u*_*b*_ = *f* ^(*b*)^(*x*), *v*_*b*_ = *f* ^(*b*)^(*x*). From (1) we obtain that

(18) f(b)(x∨y)=f(b)(x)∨f(b)(y)=ub∨vb,     that is  ⁡(x∨y,ub∨vb)∈f(b).

Take *c* ∈ *P*(*L*) such that *c*≱*a*, and then *a*≰*b*∨*c*. And take *e* ∈ *P*(*L*) such that *e* ≥ *b*∨*c* and *e*≱*a*; in this way we have (*x*, *u*_*e*_) ∈ *f* ^(*e*)^⊆*f* ^(*b*)^, (*x*, *u*_*e*_) ∈ *f* ^(*e*)^⊆*f* ^(*c*)^. From (1) we know that *u*_*b*_ = *u*_*c*_ = *u*_*e*_. Then take *u* = *u*_*c*_, *v* = *v*_*c*_; we obtain that (*x*, *u*), (*y*, *v*)∈∩{*f* ^(*b*)^ | *b* ∈ *P*(*L*), *a*≰*b*} = *f*_[*a*]_, and (*x*∨*y*, *u*∨*v*) ∈ ⋂{*f* ^(*b*)^ | *b* ∈ *P*(*L*), *a*≰*b*} = *f*_[*a*]_. Therefore

(19) $$\begin{array}{c} f_{\left[a\right]}\left(x\vee y\right)=u\vee v=f_{\left[a\right]}\left(x\right)\vee f_{\left[a\right]}\left(y\right). \end{array}$$

Similarly, it is easy to prove that *f*_[*a*]_(*x*∧*y*) = *f*_[*a*]_(*x*)∧*f*_[*a*]_(*y*). This implies that *f*_[*a*]_ : *A*_[*a*]_ → *B*_[*a*]_ is a lattice homomorphism.

(2)⇒(1). Let *a* ∈ *P*(*L*) and *x*, *y* ∈ *A* ^(*a*)^, then *A*(*x*)≰*a*, *A*(*y*)≰*a*. Since *a* is a prime element we obtain *A*(*x*)∧*A*(*y*)≰*a*. Take *b* ∈ *M*(*L*) such that *b* ≤ *A*(*x*)∧*A*(*y*) and *b*≰*a*, and then we have *x*, *y* ∈ *A*_[*b*]_⊆*A* ^(*a*)^. From (2) we know that *f*_[*b*]_ : *A*_[*b*]_ → *B*_[*b*]_ is a lattice homomorphism, so there exist *u*, *v* ∈ *B*_[*b*]_⊆*B* ^(*a*)^ such that (*x*, *u*), (*y*, *v*) ∈ *f*_[*b*]_⊆*f* ^(*a*)^ and (*x*∨*y*, *u*∨*v*) ∈ *f*_[*b*]_⊆*f* ^(*a*)^. Hence

(20) $$\begin{array}{c} f^{\left(a\right)}\left(x\vee y\right)=u\vee v=f^{\left(a\right)}\left(x\right)\vee f^{\left(a\right)}\left(y\right). \end{array}$$

Similarly, *f* ^[*a*]^(*x*∧*y*) = *f* ^[*a*]^(*x*)∧*f* ^[*a*]^(*y*); that is, *f* ^(*a*)^ : *A* ^(*a*)^ → *B* ^(*a*)^ is a lattice homomorphism.

Similar to [11], we can easily prove the following theorems.

**Theorem 39**

Let *X*, *Y* be nonempty sets, and let *A*, *B* be fuzzy sublattices of *X*, *Y*, respectively. *f* : *A* → *B* is an *L*-fuzzy mapping, if for all *b*, *c* ∈ *L*, *β*(*b*∧*c*) = *β*(*b*) ⋂ *β*(*c*), and then the following conditions are equivalent:

1. *f* : *A* → *B* is a fuzzy lattice homomorphism;
2. for all *a* ∈ *M*(*L*), *f*_(*a*)_ is a lattice homomorphism from *A*_(*a*)_ to *B*_(*a*)_.

**Theorem 40**

Let *X*, *Y* be nonempty sets, and let *A*, *B* be fuzzy sublattices of *X*, *Y*, respectively. *f* : *A* → *B* is an *L*-fuzzy mapping, if for all *a* ∈ α*(0), *f* ^[*a*]^ is a lattice homomorphism from *A* ^[*a*]^ to *B* ^[*a*]^, and then *f* : *A* → *B* is a fuzzy lattice homomorphism.

**Theorem 41**

Let *X*, *Y* be nonempty sets, and let *A*, *B* be fuzzy sublattices of *X*, *Y*, respectively. *f* : *A* → *B* is an *L*-fuzzy mapping, if for all *b*, *c* ∈ *L*, *α*(*b*∨*c*) = *α*(*b*)⋂*α*(*c*), and then the following conditions are equivalent:

1. *f* : *A* → *B* is a fuzzy lattice homomorphism;
2. for all *a* ∈ α*(0), *f* ^[*a*]^ is a lattice homomorphism from *A* ^[*a*]^ to *B* ^[*a*]^.

## 5. Fuzzy Lattice

In Definitions 16, 17, and 18, let *A* = *X*, and then the conditions are true obviously. So we can get another special case of bi-fuzzy lattice-fuzzy lattice. Now we give its definition and corresponding theorems.

**Definition 42**

Let *L* be a completely distributive lattice, *X* ≠ *∅*, and let *R* be an *L*-fuzzy relation on *X*, if *R* satisfies for any *x*, *y*, *z* ∈ *X*, there exist *s*, *t* ∈ *X* such that

(S1) *R*(*x*, *s*) ≥ *R*(*x*, *x*), *R*(*y*, *s*) ≥ *R*(*y*, *y*),
(S2) *R*(*s*, *z*) ≥ *R*(*x*, *z*)∧*R*(*y*, *z*),
(T1) *R*(*t*, *x*) ≥ *R*(*x*, *x*), *R*(*t*, *y*) ≥ *R*(*y*, *y*),
(T2) *R*(*z*, *t*) ≥ *R*(*z*, *x*)∧*R*(*z*, *y*).

then we call *s*, *t* as supremum and infimum of *x, y* with respect to *R*, respectively.

**Definition 43**

Let *L* be a completely distributive lattice, *X* ≠ *∅*, and let *R* be an *L*-fuzzy relation on *X*, if for any *x*, *y* ∈ *X*, both supremum and infimum of *x*, *y* with respect to *R* exist, and then we call *X* as a fuzzy lattice with respect to *R*.

Same to the corresponding theorems in last section,we have the following theorem.

**Theorem 44**

Let *L* be a completely distributive lattice, *X* ≠ *∅*, and let *R* be an *L*-fuzzy relation; then (1), (2), (3), (6), (7), and (8) of the following conditions are equivalent, and (4)⇒(5)⇒(1) is true.

1. *X* is a fuzzy lattice with respect to *R*.
2. For each *a* ∈ *L*∖_{0}_, (*X*, *R*_[*a*]_) is a lattice.
3. For each *a* ∈ *M*(*L*), (*X*, *R*_[*a*]_) is a lattice.
4. For each *a* ∈ *β*(1), (*X*, *R*_(*a*)_) is a lattice.
5. For each *a* ∈ β*(1), (*X*, *R*_(*a*)_) is a lattice.
6. For each *a* ∈ *α*(0), (*X*, *R* ^[*a*]^) is a lattice.
7. For each *a* ∈ α*(0), (*X*, *R* ^[*a*]^) is a lattice.
8. For each *a* ∈ *P*(*L*), (*X*, *R* ^(*a*)^) is a lattice.

## Figures and Tables

**Figure 1:**